# Differentiating influenza from COVID-19 in patients presenting with suspected sepsis

**DOI:** 10.1007/s10096-020-04109-x

**Published:** 2020-12-03

**Authors:** Valentino D’Onofrio, Eveline Van Steenkiste, Agnes Meersman, Luc Waumans, Reinoud Cartuyvels, Karlijn Van Halem, Peter Messiaen, Inge C. Gyssens

**Affiliations:** 1 Faculty of Medicine and Life Sciences, Hasselt University, Hasselt, Belgium; 2 Department of Infectious Diseases and Immunity, Jessa Hospital, Hasselt, Belgium; 3 Department of Internal Medicine and Radboud Center for Infectious Diseases, Radboud University Medical Center, Geert Grooteplein Zuid 10, 6525 GA Nijmegen, The Netherlands; 4 Emergency Department, Jessa Hospital, Hasselt, Belgium; 5 Clinical Laboratory, Jessa Hospital, Hasselt, Belgium

**Keywords:** Influenza, COVID-19, Bacterial co-infection

## Abstract

There is a need for a quick assessment of severely ill patients presenting to the hospital. The objectives of this study were to identify clinical, laboratory and imaging parameters that could differentiate between influenza and COVID-19 and to assess the frequency and impact of early bacterial co-infection. A prospective observational cohort study was performed between February 2019 and April 2020. A retrospective cohort was studied early in the COVID-19 pandemic. Patients suspected of sepsis with PCR-confirmed influenza or SARS-CoV-2 were included. A multivariable logistic regression model was built to differentiate COVID-19 from influenza. In total, 103 patients tested positive for influenza and 110 patients for SARS-CoV-2, respectively. Hypertension (OR 6.550), both unilateral (OR 4.764) and bilateral (OR 7.916), chest X-ray abnormalities, lower temperature (OR 0.535), lower absolute leukocyte count (OR 0.857), lower AST levels (OR 0.946), higher LDH (OR 1.008), higher ALT (OR 1.044) and higher ferritin (OR 1.001) were predictive of COVID-19. Early bacterial co-infection was more frequent in patients with influenza (10.7% vs. 2.7%). Empiric antibiotic usage was high (76.7% vs. 84.5%). Several factors determined at presentation to the hospital can differentiate between influenza and COVID-19. In the future, this could help in triage, diagnosis and early management. Clinicaltrial.gov Identifier: NCT03841162

## Introduction

Seasonal influenza, caused by the influenza virus, causes 4–50 million symptomatic cases in EU/EEA and approximately 500,000 cases in Belgium per year [1, 2]. Mortality can be high, with 15,000–70,000 deaths in Europe each year [1]. Influenza season is almost exclusively limited to the winter months: from November until April in the Northern hemisphere and between June and October in the Southern hemisphere [1]. Clinical features vary from uncomplicated disease with fever and a non-productive cough to severe disease with sepsis, pneumonia and sometimes myocarditis or encephalitis and even death [1].

The coronavirus disease 2019 (COVID-19) pandemic, caused by the novel coronavirus SARS-CoV-2, resulted in 9,063,320 confirmed cases in the EU/EEA until 8th November 2020. Currently, 1,250,275 patients have died in the EU/EEA [3]. In Belgium, the first case was reported on the 2nd of March 2020 [3, 4]. On the 9th of November 2020, 500,789 cases were confirmed in Belgium, with 13,055 reported COVID-19-related deaths [4]. As with influenza, clinical features can vary from asymptomatic carriage to severe pneumonia and death [3]. In contrast to influenza, patients with COVID-19 can rapidly deteriorate shortly after admission, and close monitoring is needed [5, 6].

The severity of both respiratory virus infections is partly dependent on host factors. The elderly and those with the underlying disease have higher chances for a severe disease course and are more frequently hospitalized [7, 8]. Severe viral disease presentation can be accompanied by sepsis [9]. During the next influenza season, the distinction between influenza or COVID-19-associated sepsis could be challenging. One study reporting the clinical characteristics of both diseases compared 73 COVID-19 patients with 75 H1N1 influenza patients with acute respiratory distress syndrome (ARDS). In this retrospective case-control study, the median age and the number of male patients were higher for COVID-19 patients, but SOFA score and SOFA score-adjusted mortality were higher in patients with H1N1 influenza [10]. Two studies reported that the use of a quick SOFA score was not as useful for COVID-19 patients as for patients with sepsis [11, 12]. Additionally, one study built a diagnostic model based on laboratory characteristics, which had moderate performance in differentiating influenza pneumonia from COVID-19 pneumonia [13]. Thus, there is a need for other clinical, biochemical and imaging characteristics to assess severely ill patients presenting with a viral syndrome at the Emergency Department (ED) for diagnostic and prognostic purposes.

The primary objective of this study is to compare risk factors and identify clinical, laboratory and imaging characteristics that can differentiate between influenza and COVID-19 at clinical presentation with sepsis and that could aid in the early triage and management of these patients. The secondary objective is to assess the frequency of early bacterial co-infection and its impact on the prognosis of patients with influenza and COVID-19.

## Materials and methods

### Study design, setting and study patients

A prospective observational cohort study, as part of the Fast Assay for Pathogen Identification and Characterization (FAPIC) project, was performed between February 2019 and April 2020 at Jessa Hospital, Hasselt (clinicaltrial.gov identifier NCT03841162). Adult patients presenting at the ED with suspected sepsis, defined as patients with symptoms of systemic infection for whom blood cultures were drawn, were asked to participate in the study. Patients who tested positive for influenza by PCR were included in this analysis.

During the COVID-19 pandemic, a retrospective cohort study was performed. All adult patients with SARS-Cov-2 PCR-positive test that were hospitalized for at least 24 h were included in the cohort. Patients that were suspected of sepsis and for whom blood cultures were drawn at admission (< 24 h) were included for this analysis.

In Jessa Hospital, for all patients presenting at the ED with suspected sepsis, blood cultures are performed to exclude bacteraemia. During the yearly influenza season (December–April), nasopharyngeal swabs for the detection of influenza were taken based on clinical suspicion. Since the emergence of COVID-19, SARS-Cov-2 PCR was performed according to the national case definition which was based on the WHO case definition guidance [14–16]. After the first case of COVID-19 was admitted at Jessa Hospital on the 9th of March, the FAPIC study included 16 patients until the 17th of April. The last patient in whom influenza was detected (end of the 2020 season) was included on the 5th of March 2020. The clinical laboratory of Jessa Hospital started SARS-Cov-2 PCR testing on the 5th of March 2020.

Written informed consent was obtained from all participants with influenza. No informed consent was needed for the retrospective cohort study of COVID-19 patients, according to Belgian legislation. Documented approval for both studies was obtained from the Ethics committees of Hasselt University and Jessa Hospital.

### Microbiological studies and definition of cases

Nasopharyngeal swabs for influenza detection were analysed with real-time PCR, either with the ARIES® Flu A/B & RSV assay on the ARIES instrument (Luminex Corporation) or with an in-house multiplex PCR on Quantstudie 7 flex (ThermoFisher) for the simultaneous detection of 23 respiratory pathogens. If available, lower respiratory tract samples, such as sputum, bronchial aspirates or broncho-alveolar lavage fluid, were preferred.

Nasopharyngeal swabs for the detection of SARS-CoV-2 were analysed with an in-house developed reverse-transcriptase PCR for the *E-gene* on the ARIES analyser (Luminex Corporation). In patients with a clinical suspicion based on history, laboratory and/or chest X-ray results, and a negative initial PCR test, a second nasopharyngeal swab or, if possible, a lower respiratory tract sample such as sputum, bronchial aspirates or broncho-alveolar lavage fluid, were collected after 24–48 h [17].

For all suspected pneumonia patients with chest X-ray abnormalities, urinary antigen tests for *Streptococcus pneumoniae* and *Legionella pneumophila* were performed.

Co-infections were defined according to the ECDC definitions on the surveillance of health care associated infections [18].

### Data collection

Parameters were chosen based on clinical relevance for suspected sepsis and based on literature. Relevant data were extracted from patients’ electronic medical files for all patients in both cohorts. Age, gender and comorbidities were registered. Clinical parameters at admission in the ED were collected. These parameters included body temperature, heart rate, blood pressure (systolic, diastolic and mean), oxygen saturation (SaO_2_), partial oxygen pressure (PaO_2_), Glasgow coma scale (GCS), vasopressor use and ventilation requirements. Laboratory testing at admission for suspected sepsis included white blood cell count (WBC), platelets, haemoglobin, red blood cell distribution width (RDW), C-reactive protein (CRP), creatinine, urea, lactate dehydrogenase (LDH), bilirubin, alanine aminotransferase (ALT), aspartate aminotransferase (AST), ferritin and serum lactate. To define whether patients eventually had sepsis according to sepsis-3 definitions, the SOFA score was calculated for all patients [9]. Microbiological diagnostics (pathogen identification and susceptibility) of blood cultures, of all other clinical cultures that were performed, and of virologic PCRs were collected. Data on co-infections within the first 24 h were extracted from ED discharge letters or clinical follow-up notes in the patient’s electronic medical file, and pathogens were collected from microbiology reports. Early empiric antibiotic treatment was recorded from discharge letters, from the ED and the ward or ICU, and from clinical notes made in the patient’s electronic medical file. Antibiotic use targeted at an isolated pathogen was excluded. Outcomes of patients that were collected included ICU admission, length of stay, in-hospital mortality and destination at discharge.

### Statistical analyses

Descriptive statistics were used to analyse the patient’s characteristics. Continuous data are shown as median (interquartile range). Categorical data are reported as frequency (percentages). Differences between patients were analysed with the *χ*^2^ test or Fisher’s exact test for categorical variables and with the Mann-Whitney *U* test for continuous variables. *p* values < .05 were considered statistically significant. A multivariable logistic regression model was built, where the viral infection during hospitalization was used as the outcome (influenza or COVID-19). Parameters that were statistically significant in the univariate analysis were inserted in the starting model. A backward selection based on significance level *p* < .05 was used. Odds ratios were calculated to define independent risk factors. Receiver operating curve (ROC) analysis was done to evaluate the performance of the logistic regression model to differentiate between influenza and COVID-19. Lastly, the *χ*^2^ test or Fisher’s exact test was used to analyse the difference in patient outcomes (hospital LOS, ICU admission, mortality and destination at discharge) between patients with and without co-infection. All analyses were done using SPSS version 25 (IBM, Chicago, Illinois, USA).

## Results

### Patient characteristics

In total, between the 12th of February 2019 and the 5th of March 2020, 103 patients from the FAPIC cohort with suspected sepsis on admission tested positive for influenza, 98 for influenza A and 5 for influenza B. From the 12th of March 2020 until the 12th of April, 110 patients admitted with suspected sepsis were PCR confirmed with SARS-CoV-2. No patients had an influenza and COVID-19 co-infection. Characteristics of patients, disease severity and outcomes are shown in Table 1. There were no differences in median age or gender between the two groups. Total comorbidities evaluated by the Charlson comorbidity index (CCI) were not different. However, there were significantly more COVID-19 patients with cardiac comorbidities (65.1% vs. 14.6%, *p* = .048) and hypertension (56.4% vs. 23.3%, *p* = .000). SOFA score was not different between patient groups. Eventually, 73.6% and 69.9% of patients had sepsis, respectively. Additionally, findings on chest X-ray differed, with more abnormalities for patients with COVID-19 (81.5% vs. 37.8%, *p* = .000). In the subgroup of patients with chest X-ray abnormalities, unilateral abnormalities were more frequently present for patients with influenza (31.8% vs. 59.5%, *p* = .004) and more bilateral abnormalities for patients with COVID-19 (68.2% vs. 40.5%, *p* = .004). Significantly, more patients with COVID-19 required oxygen therapy (90.9%) compared to patients with influenza (32%, *p* = .000). Lastly, all assessed outcomes were significantly different between the two groups. There were more ICU admissions (26.4% vs. 4.9%, *p* = .000), less discharges to home (84.2% vs. 89.6%, *p* = .044), more discharges to a rehabilitation centre (6.6% vs. 0%, *p* = .044), more deaths (30.9% vs. 6.8%, *p* = .000) and a longer hospital length of stay for patients with COVID-19 (8 days vs. 5 days, *p* = .000).

**Table 1:** Patient characteristics, disease severity and clinical outcomes

Variable	Total (*n* = 213)	Influenza (*n* = 103)	COVID-19 (*n* = 110)	*p* value
Age (years, median (IQR))	73 (59–83)	76 (57–84)	73 (60–82)	.676
Gender (male)	122 (57.3)	53 (51.5)	69 (62.7)	.097
Charlson comorbidity index	1 (0–2)	1 (0–2)	1 (0–3)	.970
Cardiac comorbidities	43 (20.2)	15 (14.6)	28 (65.1)	*.048*
Hypertension	86 (40.4)	24 (23.3)	62 (56.4)	*.000*
Chronic pulmonary disease	33 (15.5)	14 (13.6)	19 (17.3)	.458
Cerebrovascular disease*	20 (9.4)	6 (5.8)	14 (12.7)	.084
Renal insufficiency	37 (17.4)	14 (13.6)	23 (20.9)	.159
Liver disease	4 (1.9)	3 (75.0)	1 (25.0)	.282
Diabetes	48 (22.5)	21 (20.4)	27 (24.5)	.468
Solid malignancies	24 (11.3)	16 (15.5)	8 (7.3)	.057
Haematological malignancies	6 (2.8)	5 (4.9)	1 (0.9)	.082
SOFA score at admission (median (IQR))	2 (1–3)	2 (1–3)	2 (1–3)	.132
Sepsis (SOFA ≥ 2)	153 (71.8)	72 (69.9)	81 (73.6)	.545
Chest X-ray performed	206 (96.7)	98 (95.1)	108 (98.2)	.214
Abnormal chest X-ray	125 (58.7)	37 (37.8)	88 (81.5)	*.000*
Unilateral abnormality	50 (23.5)	22 (59.5)	28 (31.8)	*.004*
Bilateral abnormality	75 (35.2)	15 (40.5)	60 (68.2)	*.004*
Oxygen therapy	133 (62.4)	33 (32.0)	100 (90.9)	*.000*
Vasopressor use	1 (0.47)	1 (1.0)	0 (0.0)	.300
Outcomes				
ICU admission	34 (16.0)	5 (4.9)	29 (26.4)	*.000*
Length of stay (days, median (IQR))	7 (4–12)	5 (2–10)	8 (6–14)	*.000*
Destination at discharge				*.042*
Home	150 (87.2)	86 (89.6)	64 (84.2)	
Rehabilitation centre	5 (2.9)	0 (0.0)	5 (6.6)	
In-hospital mortality	41 (19.2)	7 (6.8)	34 (30.9)	*.000*

Numbers are presented as *N* (%) unless specified

*Cerebrovascular disease included strokes and TIA; *ICU*, intensive care unit. *p* < .05 was considered statistically significant

### Univariate analysis: clinical and laboratory characteristics

Clinical and laboratory characteristics of patients with suspected sepsis at clinical presentation are shown in Table 2. Patients with COVID-19 had a lower temperature at clinical presentation (37.5 °C) than patients with influenza (38.4 °C, *p* = .000). Furthermore, median SaO_2_ and PaO_2_/FiO_2_ for patients with COVID-19 was 91.5% and 309.52 respectively, which were both significantly lower than patients with influenza (93%, *p* = .005 and 328.57, *p* = .027, respectively). More patients with COVID-19 had hypoxemia (SaO_2_ < 90% or PaO_2_/FiO_2_ < 300) compared to patients with influenza (SaO_2_: 34.6% vs. 16.7%, *p* = .003 and PaO_2_/FiO_2_: 46.5% vs. 32.7%, *p* = .046, respectively). Median absolute leukocyte count was 6.29 × 10^9 cells/L in patients with COVID-19 and 8.44 × 10^9 cells/L in patients with influenza, which was significantly lower (*p* = .012). This difference could also be seen for an absolute neutrophil count (5.17 × 10^9 cells/L vs. 6.35 × 10^9 cells/L, *p* = .049) but not for absolute lymphocyte count (0.63 × 10^9 cells/L vs. 0.63 × 10^9 cells/L, *p* = .960). Overall, 58.2% of patients with COVID-19 and 67.7% of patients with influenza had lymphopenia (*p* = .354). Other biochemical parameters that were significantly higher in patients with COVID-19 than in patients with influenza were LDH, ALT, AST, bilirubin and ferritin (370 U/L vs. 230 U/L, 29 U/L vs. 20 U/L, 40 U/L vs. 26 U/L, 0.52 mg/dL vs. 0.42 mg/dL and 1000 ng/mL vs. 290 ng/mL, respectively; *p* = .000 for each and *p* = .021 for bilirubin).

**Table 2:** Clinical and laboratory characteristics of patients with suspected sepsis and confirmed viral aetiology at clinical presentation

Variable	Total (*n* = 213)	Influenza (*n* = 103)	COVID-19 (*n* = 110)	*p* value
Temperature (°C)	38 (37.2–38.6)	38.4 (37.9–38.9)	37.5 (36.5–38.1)	*.000*
Heart rate (beats/min)	94 (79–105)	96 (79–107)	90 (78.5–102.25)	.062
Systolic blood pressure (mmHg)	131 (116–146)	131 (116–144)	131 (115–150)	.781
Mean arterial pressure (mmHg)	92.67 (81–101)	91 (83–100)	95 (78–103)	.344
SaO_2_ (%) at ambient air	92 (89–95)	93 (91–96)	91.5 (88–94)	*.005*
PaO_2_/FiO_2_ ratio	328.57 (285.71–376.19)	328.57 (295.24–409.52)	309.52 (376.19–261.9)	*.027*
Glasgow coma scale	15 (15–15)	15 (15–15)	15 (15–15)	.895
Serum lactate (mmol/L)^a^	1.47 (1.18–1.96)	1.45 (1.09–1.93)	1.5 (1.2–2)	.357
CRP (mg/L)^b^	75.5 (34.25–130)	47 (22.75–99)	93 (61.5–152.5)	*.000*
Absolute leukocyte count (× 10^9/L)^b^	7.26 (5.16–9.93)	8.44 (6.02–10.4)	6.29 (4.92–9.24)	*.012*
Absolute lymphocyte count (× 10^9/L)^c^	0.63 (0.41–0.94)	0.63 (0.39–0.98)	0.63 (0.42–0.91)	.960
Absolute neutrophil count (× 10^9/L)^c^	5.7 (3.95–8.15)	6.35 (4.26–8.63)	5.17 (3.8–7.62)	*.049*
Haemoglobin (g/dL)^b^	13.25 (11.9–14.3)	13.1 (11.65–14.03)	13.5 (12.2–14.63)	*.039*
Platelets (× 10^9/L)^a^	182 (141–242)	194 (144–245.5)	174 (139.75–240.5)	.364
Creatinine (mg/dL)	1.05 (0.8–1.37)	1.04 (0.8–1.35)	1.08 (0.8–1.38)	.750
LDH (U/L)^d^	280 (220–400)	230 (190–290)	370 (262.5–450)	*.000*
ALT (U/L)	26 (17–36)	20 (14–31)	29 (20.75–42)	*.000*
AST (U/L)	33 (24–48.5)	26 (20–38)	40 (30–55)	*.000*
Bilirubin (mg/dL)^b^	0.47 (0.33–0.68)	0.42 (0.3–0.7)	0.52 (0.38–0.67)	*.021*
Ferritin (ng/mL)^e^	540 (240–1300)	290 (130–560)	1000 (515–1650)	*.000*
D dimers (mg/L)^f^	0.88 (0.52–1.49)	0.99 (0.52–1.68)	0.87 (0.51–1.47)	.740

Values are presented as median (IQR). Legend: *SaO*_*2*_, oxygen saturation; *PaO*_*2*_*/FiO*_*2*_ *ratio*, ratio of partial oxygen pressure (calculated from SaO2) to the fraction inspired air; *CRP*, C-reactive protein; *LDH*, lactate dehydrogenase; *ALT*, alanine aminotransferase; *AST*, aspartate aminotransferase. *p* < .05 was considered statistically significant

^a^*n* = 211(missing n = 2)

^b^*n* = 212 (missing n = 1)

^c^*n* = 209 (missing *n* = 4)

^d^*n* = 206(missing *n* = 7)

^e^*n* = 205 (missing *n* = 8)

^f^*n* = 124 (missing *n* = 89)

### Multivariable analysis: prediction of COVID and influenza

**Fig. 1:**
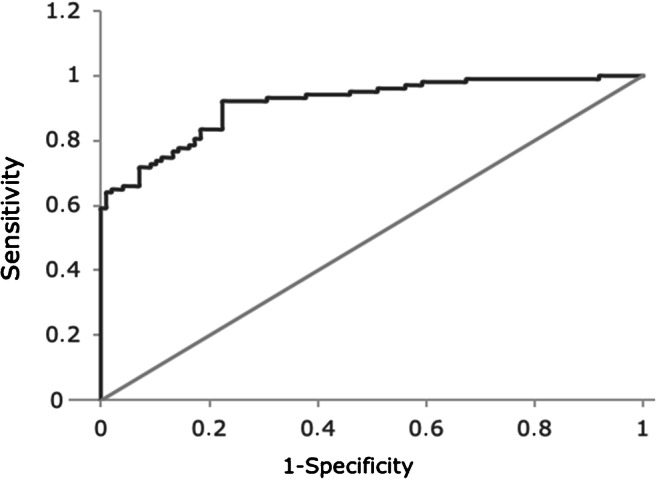
Receiver operating curve of the logistic regression model to differentiate between influenza and COVID-19

In Table 3, the multivariable logistic regression analysis of the association of clinical, laboratory and imaging characteristics with influenza or COVID-19 is presented. All variables that were significant in the univariate analysis were included in the multivariable regression model, and a backward selection was performed. In total, 14 cases were not inserted in the model since they had one or more missing values. The presence of hypertension was predictive for COVID-19 (OR 6.550 [95% CI, 2.590–16.565]). Abnormalities on chest X-ray, both unilateral and bilateral, were also predictive for COVID-19 (OR 4.764 [95% CI, 1.743–13.023] and OR 7.916 [95% CI, 3.858–21.925] respectively). Other factors that could differentiate influenza from COVID-19 were temperature, absolute leukocyte count, LDH, ALT, AST and ferritin. Patients with COVID-19 had lower temperature (OR 0.535 [95% CI, 0.343–0.833]), lower absolute leukocyte count (OR 0.857 [95% CI, 0.761–0.976]) and lower AST levels (OR 0.946 [95% CI, 0.917–0.976]). On the other hand, higher levels of LDH (OR 1.008 [95% CI, 1.004–1.012]), ALT (OR 1.044 [95% CI, 1.012–1.077]) and ferritin (OR 1.001 [95% CI, 1.000–1.002]) were predictive of COVID-19. The ROC of the model is shown in Fig. 1. ROC analysis of the logistic regression model showed an area under the curve (AUC) of 0.915 (*p* = .000; [95% CI, 0.877–0.953]). At a cutoff value of 0.49, the sensitivity was 83.5% and the specificity was 81.6%. The PPV and NPV of the logistic regression model were 82.6% and 82.5% respectively.

**Table 3:** Multiple logistic regression analysis of the association between clinical, laboratory and imaging characteristics with influenza or COVID-19

Variable	Estimate (S.E.)	OR (95% CI)	*p* value
(Intercept)	20.911 (8.588)		
Hypertension	1.879 (0.473)	6.550 (2.590–16.565)	.000
Unilateral abnormality on chest X-ray	1.561 (0.513)	4.764 (1.743–13.023)	.002
Bilateral abnormality on chest X-ray	2.069 (0.520)	7.916 (2.858–21.925)	.000
Temperature	− 0.626 (0.226)	0.535 (0.343–0,833)	.006
Absolute leukocyte count	− 0.154 (0.061)	0.857 (0.761–0.965)	.011
LDH	0.008 (0.002)	1.008 (1.004–1.012)	.000
ALT	0.043 (0.016)	1.044 (1.012–1.077)	.007
AST	− 0.055 (0.016)	0.946 (0.917–0.976)	.001
Ferritin	0.001 (0.000)	1.001 (1.000–1.002)	0.006

Legend: *LDH*, lactate dehydrogenase; *ALT*, alanine aminotransferase; *AST*, aspartate aminotransferase

### Bacterial co-infections

In Table 4, the type of co-infections and causative pathogens is shown. There were more patients with influenza diagnosed with bacterial co-infections during the first 24 h after the clinical presentation (10.7%) compared to patients with COVID-19 (2.7%, *p* = .011). Urinary antigen test for *Streptococcus pneumoniae* and *Legionella pneumophila* was performed in 59 patients with COVID-19 (53.6%) and in 81 patients with influenza (78.6%). *Legionella pneumophila* antigen was negative in all patients. *Streptococcus pneumoniae* antigen was not detected in any patient with COVID-19 but was positive in six patients with influenza. In Table 5, the univariate analysis evaluating the differences in clinical outcomes between all patients with early bacterial co-infections and those without is presented. There were no differences in clinical outcomes in patients with bacterial co-infection compared to patients without bacterial co-infection. Empiric antibiotic therapy was frequently started both in influenza and COVID-19 patients (76.7% vs. 84.5%, *p* = .147).

**Table 4:** The presence of bacterial co-infections in the first 24 h, with causative pathogens

Variable	Total (*n* = 213)	Influenza (*n* = 103)	COVID-19 (*n* = 110)	*p* value
Diagnosis/pathogen	14 (6.6)	11 (10.7)	3 (2.7)	*.019*
BSI	4 (1.9)	1 (1.0)	3 (2.7)	
No focus	2 (0.9)	0 (0.0)	2 (1.8)	
*Staphylococcus hominis*	1 (0.45)	0 (0.0)	1 (0.9)	
*Corynebacterium aurimucosum*	1 (0.45)	0 (0.0)	1 (0.9)	
Pulmonary focus	1 (0.5)	0 (0.0)	1 (0.9)	
*Streptococcus pyogenes*	1 (0.5)	0 (0.0)	1 (0.9)	
Intra-abdominal focus	1 (0.5)	1 (1.0)	0 (0.0)	
*Escherichia coli*	1 (0.5)	1 (1.0)	0 (0.0)	
Bacterial pneumonia	6 (2.8)	6 (5.8)	0 (0.0)	
Streptococcus pneumoniae*	6 (2.8)	6 (5.8)	0 (0.0)	
Urinary tract infection	3 (1.4)	3 (2.9)	0 (0.0)	
*Escherichia coli*	2 (0.9)	2 (1.9)	0 (0.0)	
Not microbiologically confirmed	1 (0.5)	1 (1.0)	0 (0.0)	
Intra-abdominal infection	1 (0.5)	1 (1.0)	0 (0.0)	

Numbers are presented as *N* (%)

*Confirmed by urinary antigen test for *Streptococcus pneumoniae*

**Table 5:** Univariate analysis comparing the clinical outcome of patients with sepsis of viral aetiology with and without bacterial co-infection during the first 24 h following clinical presentation

Outcome	Total	Bacterial co-infection (*n* = 14)	No bacterial co-infection (*n* = 199)	*p* value
ICU admission	34 (16.0)	1 (7.1)	33 (16.6)	.351
Destination at discharge (home)	150 (87.2)	10 (71.4)	140 (70.4)	.246
Length of stay (days) (median (IQR))	7 (4–12)	8.5 (4.5–14.25)	7 (4–11)	.637
In-hospital mortality	41 (19.2)	1 (7.1)	40 (20.1)	.235

Either influenza or COVID-19. Numbers are presented as *N* (%) unless specified

## Discussion

This study shows the clinical, laboratory and imaging differences between patients with influenza and patients with COVID-19 who were suspected of sepsis at clinical presentation to the hospital. More patients with COVID-19 had cardiac comorbidities and hypertension than patients with influenza. Overall, COVID-19 was associated with worse clinical outcomes, a higher risk of ICU admissions, longer hospital stay, higher mortality and more discharge to a rehabilitation centre. Furthermore, abnormalities on chest X-ray were more often found in patients with COVID-19, compared to patients with influenza. Early bacterial co-infections were diagnosed in less than 10% of patients, although more frequently in patients with influenza compared to patients with COVID-19. Empiric antibiotics were prescribed to three-fourths of all patients.

Cardiac comorbidities and hypertension were reported as risk factors for mortality in patients with COVID-19 [7] while these were not a risk factor for ICU admission in patients with influenza [8]. In our series, both patient groups had suspected sepsis but patients with COVID-19 had worse clinical outcomes, despite similar SOFA scores on admission. This is in contrast with a series from China comparing COVID-19 with H1N1 influenza patients who had developed ARDS [10]. ARDS patients with influenza had a higher mortality, even after adjusting for SOFA score. In a recent Dutch study, Beumer et al. reported a 23% ICU admission rate in patients with influenza during the 2015–2016 season which is higher than the 4.9% in this analysis [8]. Reasons could be differences in seasonal virulence, underlying illness, recruitment of patients or referral patterns. The presence of early bacterial co-infections did not impact the clinical outcome of patients admitted with a viral infection.

Univariate analyses in our study confirm the findings in the Chinese ARDS patients study, showing higher body temperature in patients with influenza and higher levels of CRP, LDH and AST in patients with COVID-19 [10].

The finding that patients with COVID-19 with abnormalities on chest X-ray had more often bilateral abnormalities is in accordance with previous studies [19]. The presence of abnormalities, both unilateral and bilateral, was predictive for COVID-19, but in our series of influenza patients, inclusion was not based on the presence of lung infiltrates.

In the best-fitted multivariable logistic regression model, the presence of hypertension, unilateral and bilateral chest X-ray abnormalities, lower body temperature, absolute leukocyte count and AST, and higher LDH, ALT and ferritin were able to predict COVID-19 and differentiate the infection from influenza. The model could very accurately predict COVID-19 with a sensitivity of 83.5% and a specificity of 81.6%. Luo et al. described a diagnostic model combining 18 routine biochemical tests. The model could distinguish between influenza and COVID-19 with an accuracy of 69.6% [13]. The higher accuracy of the model presented here shows that a combination of clinical, laboratory and imaging techniques results is beneficial.

Lastly, our finding that bacterial co-infections are less frequent in patients with COVID-19 is supported by a meta-analysis performed by Lansbury et al. [20]. The prevalence of 7% reported by these authors seems slightly higher than the prevalence we found, but we only assessed bacterial co-infections in the early stage of viral infection. Regarding empiric antibiotic use, the hospital policy recommended empirical antibiotics in the patients suspected of COVID-19 with sepsis based on the WHO interim guidance 13 March 2020 [21]. Early in the pandemic, empiric amoxicillin-clavulanic acid therapy was used. This explains the higher use of antibiotics in patients with COVID-19 compared to patients with influenza. This analysis provides further evidence that antibiotics should not be started for all patients with COVID-19. The WHO has adapted its recommendations for empirical antibiotics [22].

One of the strengths of this analysis is the availability of two large datasets. Both patient groups were similarly defined, and this resulted in a high number of severely ill patients included. The prospective data collection resulted in data of high quality and both cohorts had a low number of missing values. This study has some limitations. Patients with influenza were included during two consecutive seasons, while the patients with COVID-19 were included in 1 month. Second, we did not collect data on all features and risk factors that are known to be associated with COVID-19 such as obesity and coagulation abnormalities. Early in the pandemic, coagulation tests were not routinely performed in the absence of symptoms suggesting thrombotic events. Obesity is a risk factor for both severe influenza and severe COVID-19 [23, 24]. Third, we did not study the patients during the whole hospitalization period. Lastly, this is a single-centre study without an external validation in a different cohort. This limits the extrapolation of these results to other hospitals, and a prospective cohort is needed to validate our logistic regression model.

In conclusion, differences in clinical, laboratory and imaging characteristics between patients with influenza and patients with COVID-19 presenting with suspected sepsis were identified. Early bacterial co-infection in COVID-19 seems to be rare. Empirical antibiotics should be reserved for patients in whom a bacterial infection is suspected. A multivariable logistic regression model was used to define factors that can differentiate between influenza and COVID-19 in this population. In the future, these early features could help in rapidly triaging patients during the influenza season. Future studies should validate our results in larger cohorts.

## Data Availability

The datasets generated during and/or analysed during the current study are available from the corresponding author on reasonable request.
